# Recording of orofacial manifestations associated to rare diseases: development of a relational database

**DOI:** 10.1186/s13005-026-00662-6

**Published:** 2026-10-24

**Authors:** Sulamita Lemos Lima, Ricardo Armini Caldas, Marcus Paulo Amorim, João Victor Cunha Cordeiro, Ole Oelerich, Linda Daume, Johannes Kleinheinz, Marcel Hanisch, Lauren Bohner

**Affiliations:** 1 Department of Dentistry, Federal University of Santa Catarina, Florianópolis, Brazil; 2 Department of Prosthodontics and Periodontology, Piracicaba Dental School, University of Campinas (UNICAMP), Piracicaba, São Paulo, Brazil; 3 Department of Cranio-Maxillofacial Surgery, University Hospital Muenster, Waldeyerstraße 30, Muenster, 48149 Germany

**Keywords:** Rare Diseases, Signs and symptoms, Database

## Abstract

**Background:**

A rare disease is any condition that affects no more than one in every 2.000 individuals in a given population. These diseases have varied causes, which, in many cases, hinders early diagnosis and results in a poor quality of life. Due to their low incidence, the information available on these conditions is limited, highlighting the need to expand knowledge regarding rare diseases. The purpose of this study was to create a relational database containing information orofacial manifestations associated to rare diseases.

**Methods:**

Medical literature was systematically assessed to identify clinical signs and symptoms affecting the orofacial region. The findings were clustered to identify orofacial manifestations with common characteristics. In order to map the RDs and the related orofacial manifestations, the primary data source was the platform Orphanet, from which information about rare diseases, medical terminologies, and “Human Phenotype Ontology” (HPO) identifiers was retrieved. Data retrieval and wrangling were conducted in Python using the Spyder integrated development environment (IDE) within the Anaconda distribution. The relational data frame was modeled using MySQL Workbench.

**Results:**

In total, 258 oral manifestations were classified in 11 groups. From all rare diseases, 863 presented at least one orofacial manifestation. Bone/skeletal manifestations were the conditions mostly associated with rare diseases, followed by oral mucosal manifestations, dental manifestations, craniofacial anomalies, and neoplasic manifestation.

**Conclusion:**

Within the limitations of this study, it was possible to map rare diseases presenting orofacial manifestations. Further development of the database is required to provide technical and scientific support to professionals.

**Supplementary Information:**

The online version contains supplementary material available at https://doi.org/10.1186/s13005-026-00662-6.

## Background

A disease is considered rare when it presents a specific set of clinical signs and symptoms, affecting no more than one person per 2.000 individuals in a population [1–3]. Due to the heterogeneity of etiological factors and clinical manifestations, the exact number of rare diseases (RDs) remains unknown. However, it is estimated that approximately 6.500 diseases are considered rare [3].

RDs present a high diversity of clinical manifestations affecting different organs and body tissues, which can occur in a chronic, progressive, degenerative, or even disabling manner [4] These medical conditions may impact the quality of life from the affected person and caregivers, who often face physical, emotional, financial, and social challenges over time [5, 6]. Furthermore, most rare diseases have no cure, reinforcing the need of medical approaches to increase the patient’s quality of life [7, 8].

One of the main challenges when dealing with RDs is the lack of information due to their low prevalence and the variability of clinical manifestations. This situation affects not only the patients, but also healthcare professionals, who often have limited experience with these medical conditions [7, 9]. As a result, diagnosis may be delayed, occurring in some cases at the end of late childhood, after numerous consultations and incorrect diagnoses, which may increase the patient’s suffering, contributing to disease progression and worsening [10].

In this sense, dental surgeons play an essential role for early diagnosis, since clinical manifestations of RDs can affect hard and soft tissues of the orofacial region [5, 11, 12]. Additionally, dental interventions, as dental implant rehabilitation, orthodontic treatments, and conservative measures, contribute to promote well-being and to reduce the impact of chronic diseases on oral health-based quality of life [13–15].

However, scientific evidence regarding oral manifestations of rare diseases remains scarce. Consequently, dental treatment protocols are usually based on studies conducted in a healthy population, increasing the risk of inappropriate management and unpredictable clinical outcomes in patients with RDs [16, 17].

In order to overcome these limitations, the development of a database to record information about orofacial manifestations in people with RDs has been previously described [18]. However, there is a need to update the available knowledge, and to develop resources to make the information accessible to the general population. Therefore, the present study proposes the development of a relational database to record information about orofacial manifestations in people with rare diseases.

## Methods

### Definition of groups of interest

First, it was necessary to identify all possibilities of clinical signals, symptoms, and pathologies affecting the orofacial region. For this purpose, we first categorized signals and symptoms in groups. These groups were divided according to the anatomical region and tissue affected by the orofacial manifestation, as described in previous literature [18, 19]. A complete description of the groups is found in Appendix 1.

In order to map the RDs and the related orofacial manifestations, the primary data source was the platform Orphanet [20], which provides evidence-based knowledge about rare diseases, including etiology, diagnosis, treatment, and health care approaches. Each disease is assigned with an Orphacode and aligned with medical terminologies. The platform also provides information about phenotypes related to rare diseases and the respective “Human Phenotype Ontology” (HPO) identifiers [21].

Information of interest was extracted from Orphadata API, which allows the programmatic access to the database files. The corresponding JavaScript Object Notation (JSON) responses were retrieved and converted to tabular datasets in csv. format. Data retrieval and wrangling were conducted in Python using the Spyder integrated development environment (IDE) within the Anaconda distribution. Pseudocodes for data retrieval and filtering are described in Appendix 2.

### Rare diseases

We first retrieved information about rare diseases and the associated medical terminologies Orphacode, CID-11 and OMIM. Since these data were acquired separately, it was not possible to associate all medical terminologies to the disease name with this output. Therefore, in addition to the data collected on Orphadata API, a list containing all rare diseases and the respective codification was acquired from the Brazilian network RARASnet [22, 23]. A Python-Script was used to merge the data acquired from both sources in an unique .csv file containing the rare disease name, Orphacode, OMIM, and CID-11 (Appendix 2a, b).

### Oral manifestations

A data containing all phenotypes and their associated HP identifiers was retrieved in json. format from Orphadata API. In order to identify the phenotypes related to orofacial manifestations, a filtering process was applied using key-words of interest, which were established based on the group clustering previously described. Language variations were ensured by truncation and wildcard operators [24], as follows:

- “teeth”, “tooth”, “dent*”, “mouth”, “palat*”, “bone”, “ging*”, “perio*”, “xerost*”, “lip* fac*”, “orth*”, “dysg*jaw*”, “mandib*”, “disorder”, “temporomandibular”, “tongue”, “mucos*”, “tumor”, “neopl*”, “head” e “neck”.

A custom Python script was developed to query the endpoint for phenotypes related to orofacial structures and the respective HPO codes using the key-words as input (Appendix 2c). The output resulted in HPO identifiers and the preferred terms related to orofacial structures. After, the results were manually reviewed by two independent examiners (LS, MPA), in order to ensure that only orofacial manifestations were included in the final output. In case of disagreement, a third reviewer (LB) was consulted.

### Development of a relational database

Based on the previous output, it was possible to identify on Orphadata all rare diseases associated with orofacial manifestations. For this purpose, a custom Python script was developed to conduct a systematic search using the HPO identifiers as input (Appendix 2d).

Relational databases were modelled using MySQL Workbench. Orphacodes and HPO identifiers were determined as primary keys. For the exploratory analysis, views were created to associate the relation between rare diseases and respective orofacial manifestations (Appendix 3).

## Results

**Fig. 1:**
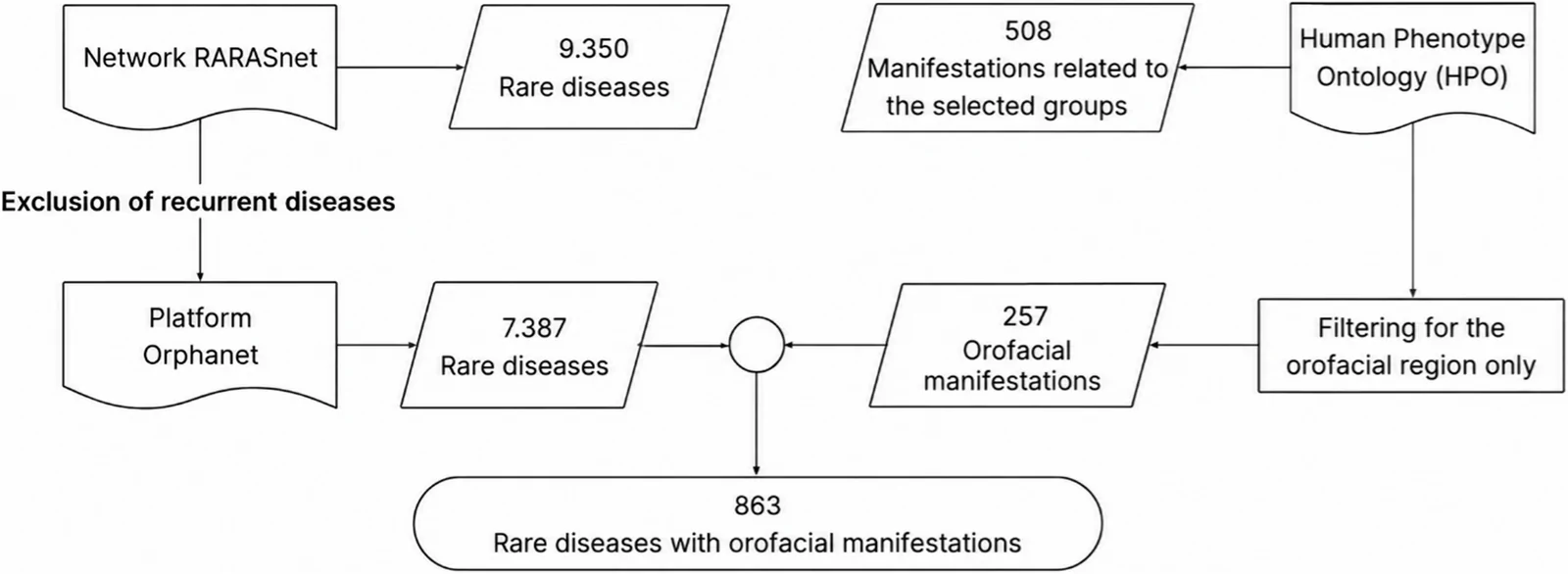
Data retrieval process

Clinical signs and symptoms affecting the orofacial region were categorized in 11 groups. Our search resulted in 257 orofacial manifestations, which were clustered according to the groups. In total, 7.387 rare diseases were identified, from which 863 presented at least one orofacial manifestation (Fig. 1). The group distribution, associated with the quantitative data of rare diseases and orofacial manifestations, is shown in Table 1. The results are available in a public repository (github.com/laurenbohner/raredisease.git).

**Table 1:** Quantitative data of rare diseases and the number of rare diseases and orofacial manifestations distributed by groups. According to the orofacial manifestation, the same rare disease can be associated with more than one group of orofacial manifestation

GROUPS OF OROFACIAL MANIFESTATIONS	NUMBER OF OROFACIAL MANIFESTATIONS		NUMBER OF RARE DISEASES	
	*n*	%	*n*	%
Bone/ skeletal manifestations	49	18.99	413	47.85
Craniofacial anomalies	54	20.93	230	26.65
Dental manifestations	59	22.86	302	34.99
Hematological manifestations	7	2.71	49	5.67
Tumoral manifestations	3	1.16	3	0.34
Neoplasic manifestations	12	4.65	103	11.93
Neurological manifestations	1	0.38	12	1.39
Oral mucosal manifestations	44	17.05	361	41.83
Periodontal manifestations	18	6.97	82	9.50
Salivary gland manifestations	2	0.77	20	2.31
Temporomandibular joint disorders	8	3.10	23	2.66

**Fig. 2:**
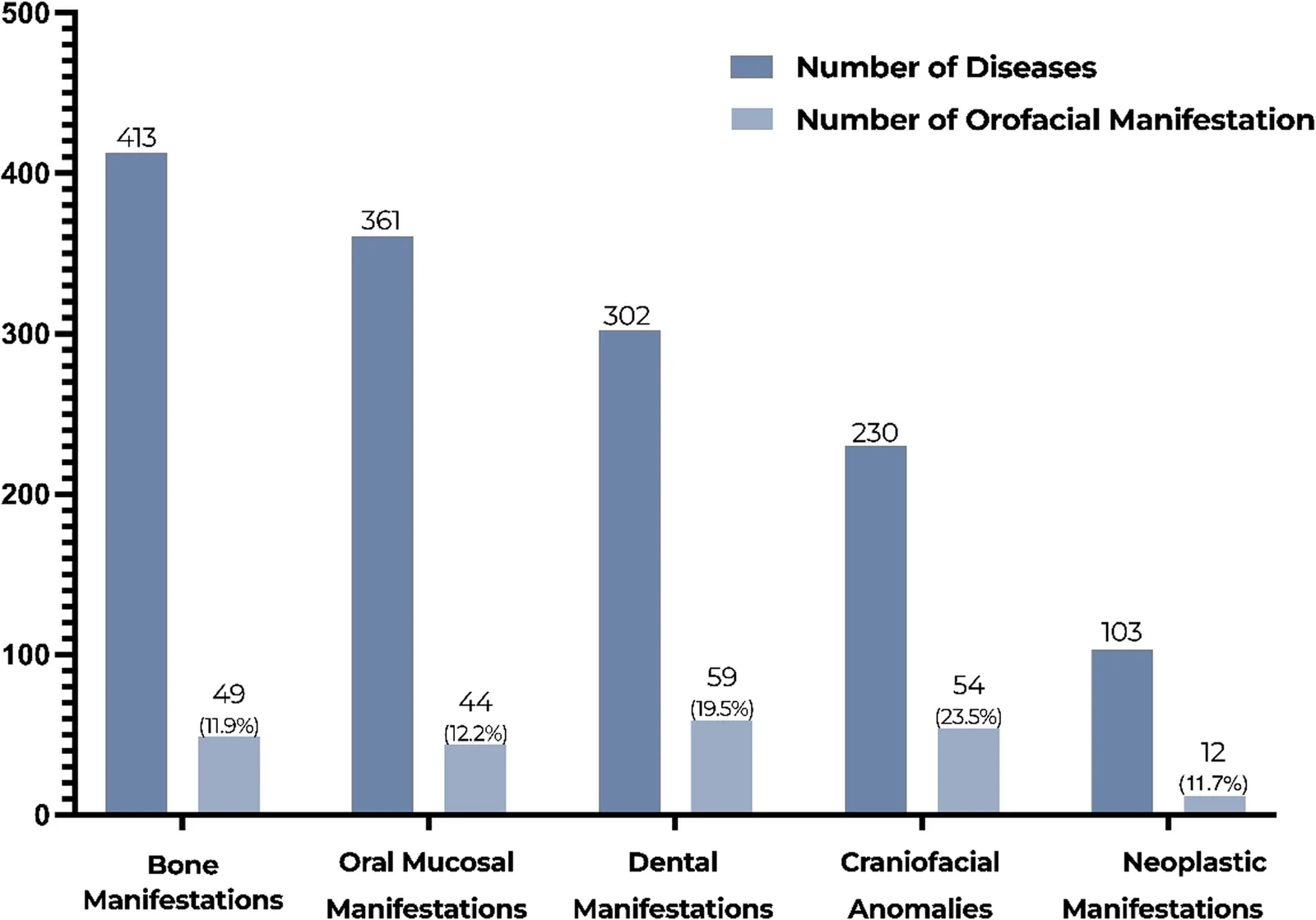
Number of rare diseases and orofacial manifestations by group

Bone/skeletal manifestations were the conditions mostly associated with rare diseases, followed by oral mucosal manifestations, dental manifestations, craniofacial anomalies, and neoplasic manifestations (Fig. 2).

**Fig. 3:**
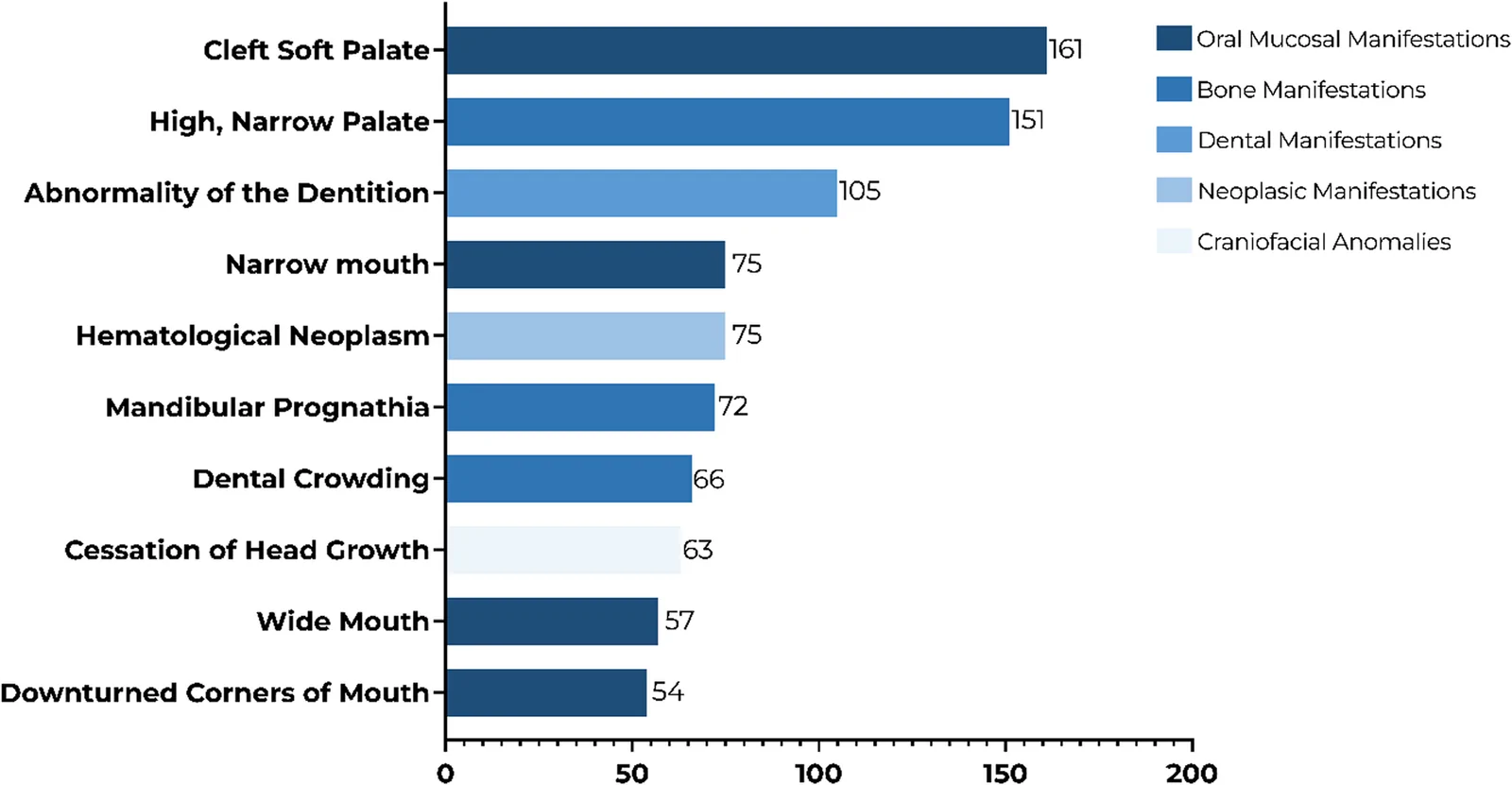
List of the oral manifestations associated with rare diseases with orofacial manifestations within their respective groups

**Fig. 4:**
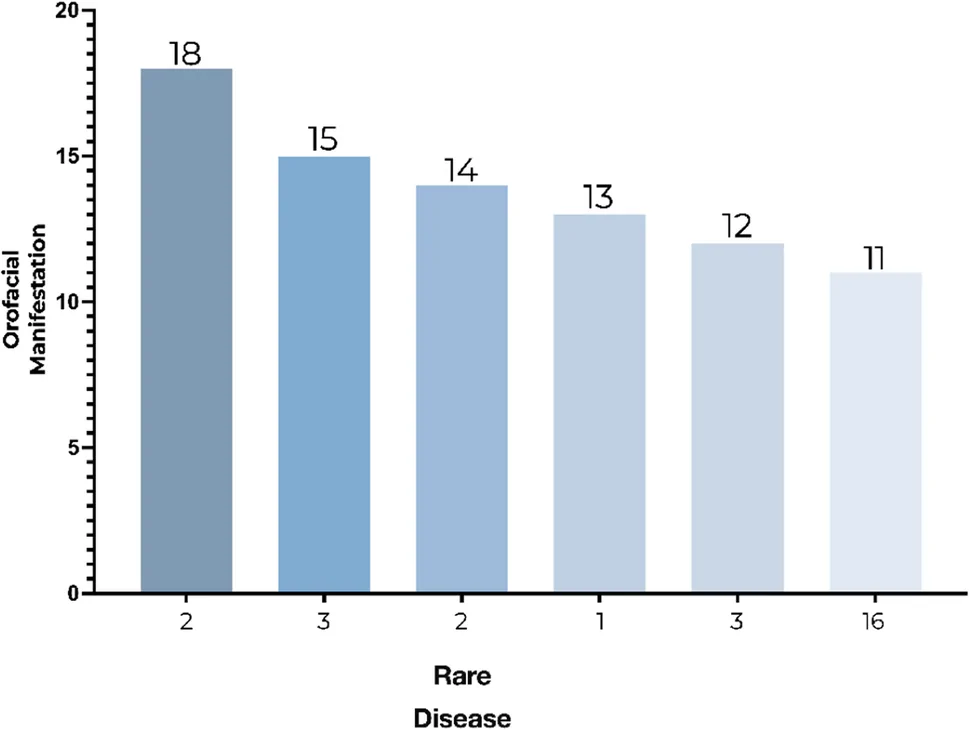
Number of rare diseases with the highest prevalence of orofacial manifestations

Among the orofacial manifestations, those mostly associated with RDs were: “Cleft soft palate”, “High, narrow palate”, “Abnormality of the dentition”, “Narrow mouth”; “Hematological neoplasm”, “Mandibular prognathia”; “Dental crowding”, “Cessation of head growth”, “Wide mouth”, and “Downturned corners of the mouth.” The remaining orofacial manifestations were associated with fewer than 50 RDs (Fig. 3). In total, 27 rare diseases presented more than 10 orofacial manifestations (Fig. 4).

## Discussion

Oral manifestations correspond to abnormalities or pathological changes affecting the hard and soft tissues of the orofacial region [5]. Rare diseases can present a wide spectrum of orofacial manifestations, which may constitute early signs or relevant indicators for their diagnosis and treatment.

In our study, approximately 11.68% of RDs were associated with orofacial manifestations. Hanisch et al. [18] claimed that approximately 15% of rare diseases may involve the orofacial region, including dental abnormalities, bone manifestations, and mucosal lesions.

Likewise, Salerno et al. [25] assessed rare genetic syndromes and reported that approximately 900 of the 5.000 described syndromes, equivalent to 18%, may present orofacial anomalies. In this context, these include a wide variety of abnormalities, such as anomalies in tooth number, changes in shape and size, structural defects in enamel and dentin, as well as malocclusions, delayed tooth eruption, alterations in the development of the maxillary and mandibular bones, and soft tissue abnormalities, such as gingival hypertrophy, ankyloglossia, and mucosal changes.

However, due to the heterogeneity of rare diseases, it is not possible to precisely define all associated orofacial manifestations. Molina-García et al. [26] conducted a systematic review to investigate oral health in patients with rare diseases. The authors reported that rheumatic conditions, with a focus on hypogammaglobulinemia, Rett syndrome, Marfan syndrome, Prader-Willi syndrome, cystic fibrosis, and Cri du Chat syndrome, presented different orofacial manifestations.

Although the dental surgeon has a significant role in diagnosing and treating orofacial manifestations in people with rare diseases, the lack of information about clinical conditions may hamper the clinical management. Friedlander et al. [27] conducted a qualitative study, interviewing patients with RDs. They reported difficulties both in maintaining oral hygiene and in accessing specialized dental services. There were frequent reports of refusal of care and uncertainty on the part of dentists due to a lack of knowledge about the manifestations associated with RDs. These findings highlight limitations in professional training and a shortage of services capable of providing appropriate care for these patients.

This is confirmed in the study by Kühne et al. [16], who conducted a study with dental surgeons to assess their knowledge and clinical approach to DRs. The results showed that most professionals had a low to moderate level of knowledge, especially regarding DRs with orofacial manifestations. In addition, a large proportion of the participants acknowledged that their knowledge was insufficient and reported not feeling prepared to manage these patients. It was also observed that professionals affiliated with university institutions demonstrated a higher level of knowledge compared to those working in clinical practice. Overall, the participants emphasized the need to expand their knowledge and receive further training in this area.

Complementing these findings, Benz et al. [17] sought to investigate knowledge and management of RDs among dental surgeons and observed that many professionals had already encountered patients with these conditions and experienced difficulties in managing them. However, most reported little interaction with colleagues to discuss cases and limited use of support networks or referrals to specialized centers. In addition, participants indicated that they primarily used information sources such as books and databases like PubMed, while the use of specific databases on RDs, such as Orphanet, was infrequent. These findings suggest limitations in access to and use of specialized resources, which may impact the proper management of clinical cases.

According to scientific evidence, there is a need for more information on RDs. Although Orphanet provides sufficient data about rare diseases, the database is focused on systemic conditions, diagnosis and treatment of conditions, whereas orofacial manifestations are briefly described. A database focused on orofacial structures may provide the dental clinicians an easy and clear information to support the dental treatment, diagnosis, and clinical decision-making when assisting patients with rare diseases. Therefore, identifying, organizing into a specific database, and classifying rare diseases is essential, as it allows for the systematization of the set of conditions studied, facilitating the analysis, comparison, and visualization of the data.

By grouping diseases with similar characteristics, it is possible to identify recurring clinical patterns, establish relationships between orofacial manifestations and broader pathological contexts, and better understand the diversity and complexity that characterize this group of diseases. Future studies should assess the influence of such information sources over the professional knowledge, such as the impact over the treatment of patients with rare diseases.

## Conclusion

Within the limitations of this study, it was possible to map rare diseases presenting orofacial manifestations in accordance to the current literature. A database validation is required to assess whether the database may provide technical and scientific support to professionals.

## Supplementary Information

Supplementary Material 1.

## Data Availability

The datasets generated and/or analyzed during the current study are not publicly available at this time, as the database is currently under development and being implemented on a publicly accessible platform. However, the datasets are available from the corresponding author upon reasonable request.

## Abbreviations

RDs: Rare diseases
HPO: Human Phenotype Ontology
JSON: JavaScript Object Notation
IDE: Spyder integrated development environment
API: Application Programming Interface
CID: International Classification of Diseases
OMIM: Online Mendelian Inheritance in Man
