# Supplementary material for: Recording of orofacial manifestations associated to rare diseases: development of a relational database
Source: Head Face Med. 2026 Oct 24;22:81. doi: 10.1186/s13005-026-00662-6 (PMC13695274; doi:10.1186/s13005-026-00662-6)
Supplement: Supplementary file 1 [file 13005_2026_662_MOESM1_ESM.docx]

Appendix 1. Groups description.

| **Groups** | **Definition** |
| --- | --- |
| **Dental manifestations** | Manifestations affecting the teeth, potentially involving their shape, structure, presence, or absence. |
| **Periodontal manifestations** | Conditions affecting the periodontium and its associated structures. |
| **Salivary gland manifestations** | Manifestations that may alter the anatomical or histological structure of the glands, cause tissue destruction, or interfere with salivary production, secretion, and composition. |
| **Temporomandibular joint disorders** | Conditions involving functional or structural alterations of the temporomandibular joint, its associated structures, and the muscles of mastication and the face. |
| **Tumoral manifestations** | Clinical, local, or systemic alterations caused by a tumor, whether benign or malignant. |
| **Neoplastic manifestations** | Alteration characterized by abnormal cell growth that may be located in the soft tissues or bones of the oral cavity. |
| **Neurological manifestations** | Alterations of the central or peripheral nervous system that affect the face and oral cavity and may present as sensory or motor disturbances. |
| **Craniofacial anomalies** | Congenital malformations affecting the bony structures of the skull and face, potentially compromising the development, shape, composition, and function of these regions. |
| **Bone/dysgnathia manifestations** | Manifestations affecting not only the anatomy of the jaw bones but also their density, metabolism, and development. This group also includes medications that interfere with the bone matrix and alterations in the relationship between the dental arches and the underlying skeletal bases. |
| **Hematological manifestations** | Clinical conditions characterized by an increased predisposition to bleeding or hematoma formation, as well as variations in blood vessel anatomy. |
| **Oral mucosal manifestations** | Anatomical variations affecting the form, structure, or function of the tissues of the lips and the tissues lining the entire oral cavity, including those affecting the dorsal and ventral surfaces of the tongue. |

Appendix 2 . Pseudocodes for retrieval and filtering using Python scripts.

|  | **Purpose** | **Pseudocode** |
| --- | --- | --- |
| **a** | Retrieval of disease names from an external API (Orphadata) for a list of clinical classification codes (Orphacode, CID-11 and OMIM). | LOAD list of input codes  INITIALIZE empty results dictionary  FOR each code:  BUILD the API request URL using the code  SEND a GET request  IF the request succeeds:  STORE the returned JSON under that code  ELSE:  STORE the error/status information  SAVE all collected results as api_cross_reference_results.json |
| b | Merging of Orphadata and RARASnet files to create an unique file associating each disease with the respective clinical classification codes | LOAD file_code.csv  LOAD file_disease.csv  STANDARDIZE relevant column names  TRIM spaces and normalize code fields  CREATE a unique set of codes from file_code.csv  FILTER file_disease rows where code is in the code set  SAVE the filtered records as matched_diseases.csv  REPORT the number of matched rows |
| **c** | Filtering of HPO terms based on the key-words of interest | DEFINE list of keywords  CONVERT wildcard terms into regular-expression patterns  LOAD phenotype_terms.json  RECURSIVELY traverse dictionaries and lists  FOR every text string found:  FOR every keyword pattern:  IF the pattern matches the text:  EXTRACT a short context snippet around the match  STORE the match under the corresponding keyword  PAD keyword result columns to equal length  CREATE table  REMOVE JSON path prefixes from displayed matches  SAVE as phenotype_keyword.csv |
| **d** | Retrieval of rare diseases associated with the selected HPO identifiers from the Orphadata API. | LOAD selected_hpo_terms.xlsx  EXTRACT non-missing HPO values  INITIALIZE empty API-results list  FOR each HPO identifier:  BUILD the phenotype API URL  SEND a GET request in English  IF successful:  READ the JSON response  ATTACH the queried HPO identifier to each returned record  APPEND records to the results list  ELSE:  REPORT the request error  NORMALIZE all JSON results into a table  SAVE as rare_diseases_by_hpo.csv |

Appendix 3. Pseudocode for the relational database in MySQLWorkbench.

| **Purpose** | **Pseudocode** |
| --- | --- |
| **1. Create and select the database** | CREATE database "Orphanet Database"  SELECT database "Orphanet Database"  CHECK active database  LIST available tables |
| **2. Create the HPO table** | CREATE table "HPO"  DEFINE HPO_ID as primary key |
| **3. Create the Disease table** | CREATE table "Disease"  DEFINE ORPHAcode as primary key  DEFINE Disease_Name as required field |
| **4. Create Disease-HPO relationships** | CREATE table "Disease_HPO"  DEFINE ORPHAcode  DEFINE HPO_ID  SET ORPHAcode + HPO_ID as combined primary key  LINK ORPHAcode to Disease table  LINK HPO_ID to HPO table  CASCADE updates and deletions when referenced records change |
| **5. Count orofacial manifestations per disease** | FOR each disease  FIND associated HPO terms  COUNT unique orofacial manifestations  RETURN ORPHAcode, Disease_Name, Number_of_Manifestations |
| **6. Count diseases per orofacial manifestation** | FOR each orofacial manifestation  FIND associated diseases  COUNT unique diseases  RETURN HPO_ID, Manifestation_Name, Number_of_Diseases |
| **7. Generate a list of manifestations for each disease** | FOR each disease  FIND associated HPO terms  COMBINE HPO_ID with Manifestation_Name  REMOVE duplicates  SORT manifestations alphabetically  CONCATENATE manifestations into one list  RETURN ORPHAcode, Disease_Name, Manifestation_List |
| **8. Count diseases by orofacial group** | FOR each Orofacial_Group  FIND associated HPO terms  FIND diseases linked to these HPO terms  COUNT unique diseases  RETURN Orofacial_Group, Number_of_Diseases |
| **11. Summarize diseases and manifestations by group** | FOR each Orofacial_Group  COUNT associated diseases  COUNT orofacial manifestations  RETURN Orofacial_Group, Number_of_Diseases, Number_of_Manifestations |
